# A Deep-Learning-Based Real-Time Detector for Grape Leaf Diseases Using Improved Convolutional Neural Networks

**DOI:** 10.3389/fpls.2020.00751

**Published:** 2020-06-03

**Authors:** Xiaoyue Xie, Yuan Ma, Bin Liu, Jinrong He, Shuqin Li, Hongyan Wang

**Affiliations:** ^1^College of Information Engineering, Northwest A&F University, Yangling, China; ^2^Key Laboratory of Agricultural Internet of Things, Ministry of Agriculture and Rural Affairs, Northwest A&F University, Yangling, China; ^3^Shaanxi Key Laboratory of Agricultural Information Perception and Intelligent Service, Northwest A&F University, Yangling, China; ^4^College of Mathematics and Computer Science, Yan’an University, Yan’an, China; ^5^Ningxia Smart Agricultural Industry Technology Collaborative Innovation Center, Yinchuan, China; ^6^West Electronic Business Co., Ltd., Yinchuan, China

**Keywords:** grape leaf diseases, object detection, deep learning, convolutional neural networks, feature fusion

## Abstract

Black rot, Black measles, Leaf blight and Mites of grape are four common grape leaf diseases that seriously affect grape yield. However, the existing research lacks a real-time detecting method for grape leaf diseases, which cannot guarantee the healthy growth of grape plants. In this article, a real-time detector for grape leaf diseases based on improved deep convolutional neural networks is proposed. This article first expands the grape leaf disease images through digital image processing technology, constructing the grape leaf disease dataset (GLDD). Based on GLDD and the Faster R-CNN detection algorithm, a deep-learning-based Faster DR-IACNN model with higher feature extraction capability is presented for detecting grape leaf diseases by introducing the Inception-v1 module, Inception-ResNet-v2 module and SE-blocks. The experimental results show that the detection model Faster DR-IACNN achieves a precision of 81.1% mAP on GLDD, and the detection speed reaches 15.01 FPS. This research indicates that the real-time detector Faster DR-IACNN based on deep learning provides a feasible solution for the diagnosis of grape leaf diseases and provides guidance for the detection of other plant diseases.

## Introduction

China is a modern agricultural country with more than 2000 years of history in grape planting. At present, China has the largest grape export in the world. At the same time, grape juice, raisins, wine, and other grape products also have great commercial value. However, severe diseases take a great toll on yield and quality during the growing process of grapes, especially in rainy areas and periods. Thus, timely and effective detection of grape leaf diseases is a vital means to ensure the healthy growth of grapes.

Traditionally, the diagnosis of plant leaf diseases relies on trained experts performing visual inspection (Dutot et al., 2013), which usually leads to high cost and a large risk of error. With the rapid development of artificial intelligence, machine learning methods have been applied to plant disease detection to make it more intelligent. Researchers began to apply machine learning algorithms to plant disease diagnosis, such as support vector machines (SVM) and K-means clustering (Es-saady et al., 2016; Mwebaze and Owomugisha, 2016; Padol and Yadav, 2016; Qin et al., 2016; Islam et al., 2017; Dickinson et al., 2018; Tian et al., 2019). However, because of the complex image preprocessing and feature extraction, these methods still have low detection efficiency. In recent years, the convolutional neural network (CNN) has been developed as an end-to-end deep learning approach, they take full advantage of image big data and discover the discriminative features directly from original images, avoiding complicated image preprocessing and reducing the memory footprint. Inspired by the breakthroughs of CNNs in pattern recognition, using CNNs to identify early plant leaf diseases has become a new focus of smart agriculture. In (Fuentes et al., 2017; Liu et al., 2017; Lu Y. et al., 2017; Ramcharan et al., 2017; Boulent et al., 2019; Bresilla et al., 2019; Polder et al., 2019; Saleem et al., 2019; Zhu et al., 2019), CNNs were mainly used to diagnose crop diseases. Nevertheless, many difficulties remain in realizing the real-time detection of grape leaf diseases due to the following characteristics of grape leaf diseased spots. Multiple small and dense diseased spots may occur on the same leaf, which are usually of various shapes. Moreover, environmental factors and shielding of other leaves also affect the detection of grape leaf diseases.

To overcome these problems, this article proposes a deep-learning-based detector based on improved CNNs to monitor grape leaf diseases in real-time. The main contributions of this article are summarized as follows:

• A grape leaf disease dataset (GLDD) is established. The GLDD provides a necessary guarantee for the generalization ability of the model. First, to improve the practicability of the model, images of diseased grape leaves with simple backgrounds in the laboratory and complex backgrounds in the grapery are collected. Furthermore, to prevent the CNN overfitting problem, the dataset is expanded via digital image processing technology to form the GLDD for providing sufficient training disease images.
• A real-time detection model for grape leaf diseases, Faster DR-IACNN, is proposed. By introducing the Inception modules and SE-block, the backbone network ResNet is modified to obtain a novel pre-network, named INSE-ResNet. Through upsampling and downsampling, the double-RPN structure is designed and achieves stronger feature extraction ability of small diseased spots. The proposed Faster DR-IACNN model improves the extraction ability of multiscale diseased spots and the detection speed of ResNet, reducing the depth and increasing the width of the neural network.
• The deep CNN is first applied to real-time detection of grape leaf diseases. The proposed end-to-end real-time detector based on deep learning can automatically extract the features of grape leaf diseases and detect the four common diseases of grape leaves efficiently. At the same time, this method can also detect a variety of diseases in the leaves at one time.

The experimental results show that the mean Average Precision of Faster DR-IACNN is 81.1%, which is 2.3% higher than that of Faster R-CNN, and the detection speed reaches 15.01 FPS. The experiments indicate the deep-learning-based detector exhibits higher detection precision and can satisfy the actual demand for real-time detection in graperies.

The rest of the article is organized as follows: section “Related Work” introduces and summarizes the related work. The GLDD is introduced in section “Generating Grape Leaf Disease Dataset.” In section “Detection Model of Grape Leaf Diseases” describes the detection model of grape leaf disease in detail. In section “Experimental Evaluation,” an evaluation of the experimental performance and analyses of the experimental results are presented. Finally, section “Conclusion” summarizes this article.

### RELATED WORK

With the development of artificial intelligence, deep learning has made breakthroughs in computer vision. It has been widely utilized to identify plant diseases and is a satisfying alternative for the classification of plant diseases. In Wang et al. (2012), proposed a grape disease recognition method based on principal component analysis and backpropagation networks. The dataset of grape diseases includes grape downy mildew and grape powdery mildew, and the prediction accuracy was up to 94.29%. In Sannakki et al. (2013), came up with a method to diagnose two types of grape diseases. Using thresholding and anisotropic diffusion to preprocess images and K-means clustering to segment disease spots, the method achieved high training accuracies when using hue features. In Mohanty et al. (2016), trained two deep learning models (AlexNet and GoogLeNet) to identify 14 crop species and 26 diseases. By examining two types of training mechanisms, three dataset types and five types of training-testing set distributions, they achieved an accuracy of 99.35%. In Lu J. et al. (2017), proposed an in-field wheat disease diagnosis system that has since been implemented in a mobile app to help agricultural disease diagnosis. By implementing two different frameworks VGG-FCN-VF16 and VGG-FCN-S, they obtained mean recognition accuracies of 97.95 and 95.12%, respectively, on WDD2017, demonstrating further improvement over the accuracies of 93.27 and 73.00% obtained using traditional CNN frameworks. In Yu et al. (2019), reported several deep CNN models for weed detection in bermudagrass turfgrasses and demonstrated that VGGNet achieved a high score at the detection of three common diseases in turfgrasses, while DetectNet could better detect disease in annual bluegrass. Based on these results, they proposed a DCNN-based recognition system for weed control. In Ferentinos (2018), applied five basic CNN architectures to an open database of 87,848 images including 25 plant species of 58 distinct classes, with the best performance reaching a recognition accuracy of 99.53%. Though deep CNNs have made great achievements in plant disease classification, real-time detection of diseases during the growth of the plant is more essential in order to control the diseases effectively at an early time.

Many researchers have begun to study how to detect plant leaf disease precisely through deep learning methods. In Rançon et al. (2018), provided a recognition and detection method based on grape leaf Esca symptoms during summer. In experiments, they compared SIFT encoding with pretrained deep learning feature extractors and implemented the MobileNet network on the ImageNet database to get a classification accuracy of 91%. Then, they combined the classification network with a one-stage detection network (RetinaNet) to obtain the best Esca AP of 70%. In Jiang et al. (2019), presented a new network architecture named INAR-SSD based on VGGNet and Inception construction. They applied the architecture to the apple leaf disease in the detection and reached 78.8% mAP. In Fuentes et al. (2018), designed a Refinement Filter Bank framework for tomato plant diseases and pests to solve the problem of false positives and class unbalance based on deep convolution neural networks. The system consists of three units—a primary diagnosis unit, a secondary diagnosis unit, and an integration unit—and the mAP was 13% greater than the best result based on Faster R-CNN. It is more efficient to detect plant leaf disease and easier to obtain higher accuracy when using a novel deep learning approach based on CNNs. According to these studies, CNNs have made a great contribution to the identification and detection of plant diseases. Unfortunately, there are no suitable CNN models for the real-time detection of grape leaf diseases, which would have high practical value in grape planting. Thus, a real-time detector based on Faster R-CNN for grape leaf diseases is proposed in this article.

## Generating Grape Leaf Disease Dataset

**FIGURE 1:**
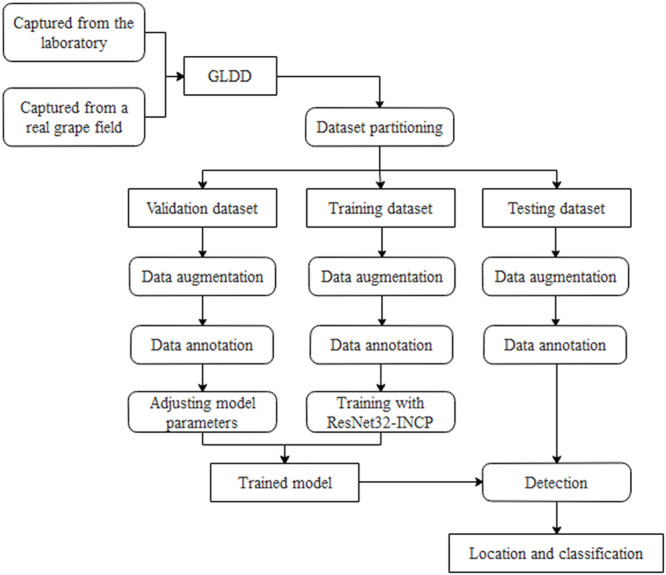
Flow chart of grape leaf disease detection.

Details of grape leaf disease detection are shown in Figure 1. First, the original grape leaf disease images are acquired from the laboratory and a real grapery. Then, the original grape leaf disease images are expanded by data argumentation operations and further refined by expert annotation. Finally, the dataset is divided into three parts: the training set is used to train the Faster DR-IACNN model, the validation set is used to adjust the parameters and evaluate the model, and the testing set is used for verifying the generalization of the model.

### Grape Leaf Disease Images Collection

Since there are few available datasets of grape leaf diseases for disease detection, many human and material resources have made significant contributions to collecting and building GLDD. Grape plants suffer from diseases in different seasons, temperatures, and humidity. For instance, Black rot causes severe damage to the grape industry in continuous hot and humid weather, but it rarely occurs in dry summer. Grape Leaf blight is extremely serious in September when the tree is weak, the temperature is low, and rain is frequent. Considering the above situations, the disease images in the GLDD were collected under various climate conditions to make the GLDD widely used. Apart from capturing images manually, the other disease images in the dataset were collected from Wei Jiani Chateau, Yinchuan, the Ningxia Hui Autonomous Region, China.

A total of 4,449 original images of grape leaf diseases were obtained, they contain four disease categories: Black rot (a fungal disease caused by an ascomycetous fungus), Black measles (also named Esca, caused by a complex of fungi such as Phaeoacremonium), Leaf blight (a common grape leaf disease caused by a fungus), and Mites of grape (caused by parasitic infestation of rust ticks). There are two reasons for choosing these four types of grape leaf diseases: first, some of the diseased spots cannot be distinguished visually, but it is easy for CNNs to extract features. Moreover, the occurrence of these diseases causes great losses to the grape industry.

**FIGURE 2:**
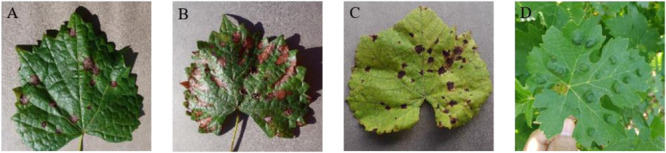
Four common types of grape leaf diseases. **(A)** Black rot. **(B)** Black measles. **(C)** Leaf blight. **(D)** Mites of grape.

Figure 2 shows typical images of four types of grape leaf diseases in GLDD. It can be intuitively observed that the four diseased spots on the grape leaves have similarity and diversity: the disease effects caused by the same disease with similar natural conditions are basically the same, while the characteristics of diseased spots caused by different diseases are usually various. Leaf infected by Black rot appears reddish-brown and have nearly round small spots that expand into the large gray spots with brown edges in the later period. The diseased spots of Black measles resemble tiger stripes, which are reddish-brown bands of necrosis. The characteristic lesions of Leaf blight are irregular, with dark red to brown spots appearing at first, followed by black spots. Mites of grape are caused by parasitic rust ticks and feature bubble-like uplift on the leaf surface. These observations are helpful for the real-time diagnosis and detection of various grape leaf diseases using deep CNNs.

The collected dataset has three following features: first, multiple diseased spots of different diseases may simultaneously appear on the same leaf. Second, many images contain complicated backgrounds of interfering spot detection, which guarantees the high generalization of Faster DR-IACNN. Finally, all images in the dataset are manually annotated by reliable experts.

### Image Augmentation

Due to the insufficient disease images, neural networks excessively obtain the information of the training set, leading to the overfitting problem in the training process of CNNs. Hence, data augmentation technology is used to simulate real-life interference, which plays an important role in the model training stage. As more images are generated via data augmentation, the model can learn as many different patterns as possible during the training, avoiding the overfitting problem and achieving better detection performance in practice.

**FIGURE 3:**
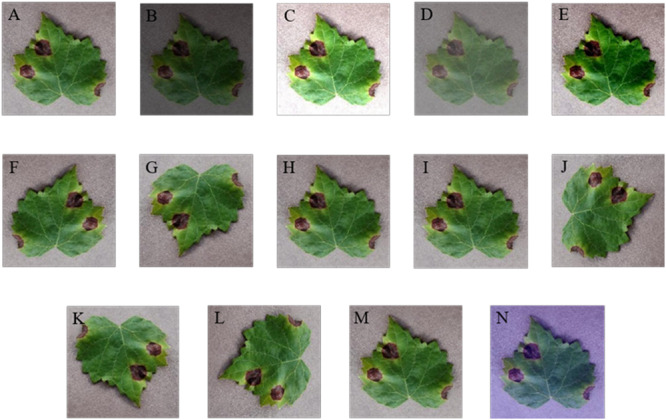
Data augmentation of grape leaf disease images. **(A)** Original image; **(B)** low brightness; **(C)** high brightness; **(D)** low contrast; **(E)** high contrast; **(F)** vertical flip; **(G)** horizontal flip; **(H)** low sharpness; **(I)** high sharpness; **(J)** 90° rotate; **(K)** 180° rotate; **(L)** 270° rotate; **(M)** Gaussian noise; **(N)** PCA jittering.

In this section, several digital image processing technologies are applied to data augmentation operations. Considering the effects of weather factors on the image intensity, interference of brightness, contrast, and sharpness are implemented. The variety in the relative shooting position of camera and diseased leaf is simulated via rotation (including 90, 180, and 270°) and symmetry (including vertical and horizontal symmetry). Gaussian noise is used to imitate the influence of equipment factors. Moreover, PCA jittering is used to expand the original dataset as well to simulate the real acquisition environment and increase the diversity and quantity of the grape leaf diseases training images. Thus, the GLDD is formed via expanding the original dataset by 14 times. Figure 3 presents a grape leaf disease image example generated through image augmentation technology.

### Image Annotation

Image annotation is a crucial step in building the dataset; it is used to mark out the location and category of diseased spots in infected leaves. In this section, a tool has been developed to annotate images through rectangular bounding boxes. Using the annotation tool and the knowledge of experienced agriculture experts, areas of diseased spots in the image can be accurately labeled. When the annotation is complete, an XML file is generated for each image, which includes the types of diseased spots and their locations.

**FIGURE 4:**
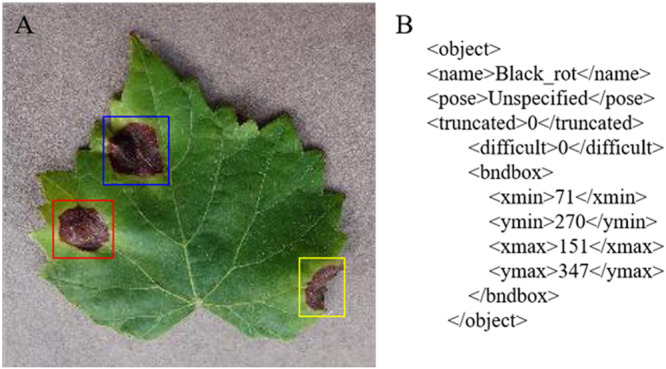
Annotation of the GLDD. **(A)** Annotated image. **(B)** XML file fragment of Black rot disease.

Take an image of Black rot as an example. The annotated image in Figure 4A shows the infected areas surrounded by red, blue, and yellow boxes. Figure 4B is a fragment of the generated XML file, in which the disease name of Black rot is described and the location of diseased spots is determined by upper left and lower right coordinates of the red box.

Due to the limitation of manual annotation and the annotation tool, inevitable random errors will occur in image annotation process. In order to reduce the influence of the errors on subsequent experiments, the image labels have been checked repeatedly. Moreover, the probability of such errors is too small to affect a large number of datasets, which can be ignored.

## Detection Model of Grape Leaf Diseases

**FIGURE 5:**
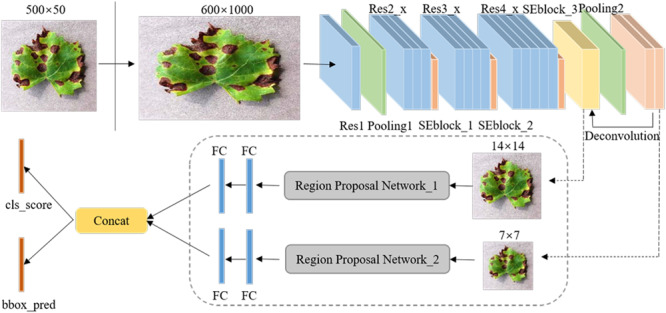
The overall structure of the Faster DR-IACNN model.

Figure 5 shows the overall framework of the Faster DR-IACNN model for detecting four typical grape leaf diseases. The DR-IA means double-RPN with Inception module and Attention structure, which contains all the characteristics of our model. The proposed Faster DR-IACNN consists of three parts: (1) a pre-network for extracting disease image features. The pre-network, namely, INSE-ResNet, includes residual structure, inception modules and SE-block. The backbone ResNet is designed for extracting images’ feature information, while the inception modules and SE-block aim to widen the receptive field and obtain multiscale features. (2) A Region Proposal Network (RPN) for locating objects. After the processing of pre-network, feature maps are sent to the RPN. In this part, the diseased spots are located and predicted by bounding boxes. (3) Fully connected layers for classification and regression. In this part, categories and scores are calculated through fully connected layers. All the information is fused in the concatenation layer. Finally, the class scores and prediction boxes are output.

### Multiscale Feature Extraction of Diseased Spots

#### Backbone Networks for Extracting Features

Due to the specialty of Black rot and Leaf blight with small and dense diseased spots, a variety of backbone networks, such as AlexNet, VGGNet, and ResNet, were experimented with and analyzed, and ResNet has been found to be the most suitable backbone network. According to the characteristics of grape leaf diseased spots, ResNet34 has a high recognition accuracy for the GLDD. Therefore, ResNet34 was selected as the pre-network of the detection model. ResNet with residual learning enables the network structure to be further deepened without the disappearance of the gradient (He et al., 2016), which solves the degradation problem of deep CNNs and fits for the small diseased spots. In addition, it is easy to optimize and achieve high accuracy in classification.

**FIGURE 6:**
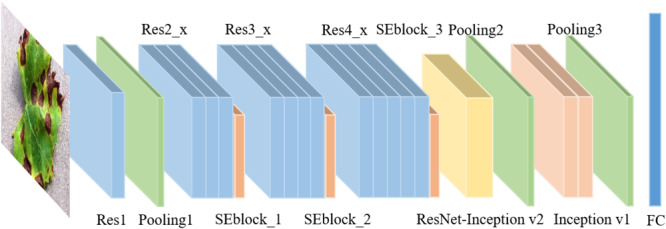
Structure of INSE-ResNet.

Table 1 lists the detailed parameters of the adjusted ResNet34, named INSE-ResNet, and Figure 6 shows the structure of INSE-ResNet. The first several layers of CNNs usually learn low-level features such as color and edges (Zeiler and Fergus, 2013), and the deeper layers extract complete and discriminative features. Thus, Res1 to Res3 of ResNet34 are completely retained. Meanwhile, the article applies Squeeze-and-Excitation Blocks in the tail of ResNet blocks. The Res_4f layer is removed, and the Res_4e layer is replaced with Inception-ResNet-v2 module to enhance the multiscale feature extraction ability of the pre-network. To fix the input size of the following-up network, the feature map is adjusted to the size of 14 × 14 through the RoI pooling layer. In the subsequent network, the Res5 layer is replaced with two Inception-v1 modules. The final output is the concatenation of the category and location losses.

**TABLE 1:** The related parameters of INSE-ResNet model.

**Output size**	**Name**	**Related parameters (kernel size, output size, stride)**
112 × 112	Res1	Convolution, 7 × 7, 64, stride 2
56 × 56	Pool1	3 × 3 max-pooling, stride 2
	Res2_x	$\left[\begin{array}{cc} \text{Conv}3\times 3 & \text{64}\\ \text{Conv}3\times 3 & \text{64} \end{array}\right]\times 3$
	SEblock_1	FC, [16, 256]
28 × 28	Res3_x	$\left[\begin{array}{cc} \text{Conv}3\times 3 & \text{128}\\ \text{Conv}3\times 3 & \text{128} \end{array}\right]\times \text{4}$
	SEblock_2	FC, [32,512]
14 × 14	Res4_x	$\left[\begin{array}{cc} \text{Conv}3\times 3 & \text{256}\\ \text{Conv}3\times 3 & \text{256} \end{array}\right]\times \text{5}$
	SEblock_3	FC, [64, 1024]
	Inception-ResNet-v2	As in Figure 8A
7 × 7	Pool2	3 × 3 max-pooling, stride 2
	Inception_5a	As in Figure 8B
	Inception_5b	As in Figure 8B
1 × 1	Pool3	7 × 7 average-pooling, stride 1
	Softmax	5

#### High Semantic Feature Extraction Modules for Diseased Spots

**FIGURE 7:**
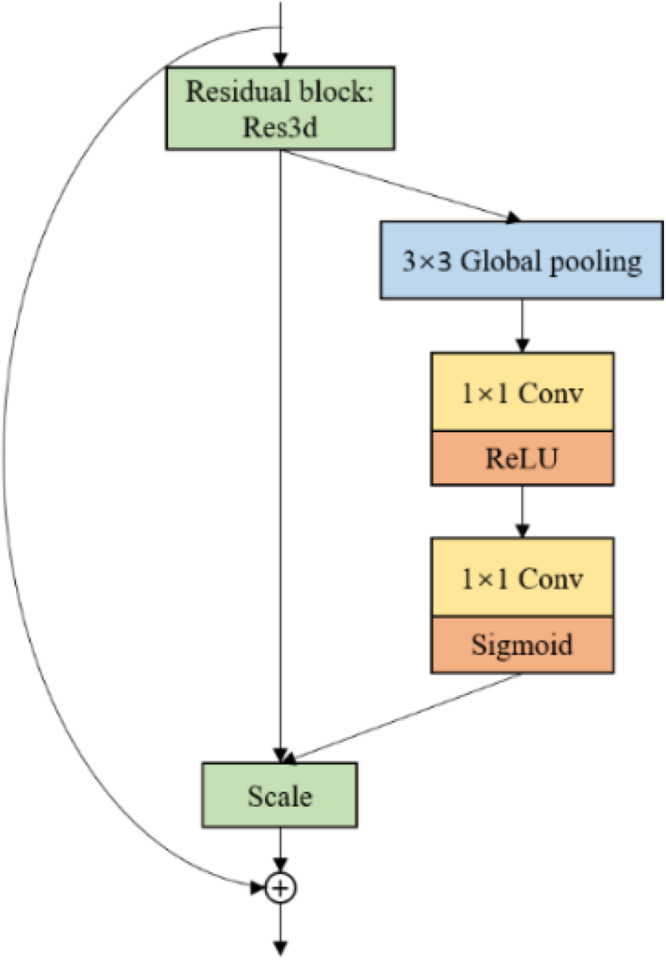
Squeeze and Excitation module.

Considering GLDD includes numerous complex background images, the network needs to focus on diseased spots, instead of the background. Thus, the article introduces Squeeze-and-Excitation Blocks (Hu et al., 2018). The SE-blocks learn the feature weights via the loss, such that the available feature map with diseased spots has a large weight, and the invalid or tiny feature map has a lightweight. Figure 7 shows the SE block structure, which stacks 3 × 3 average pooling layers and 1 × 1 convolution layers.

**FIGURE 8:**
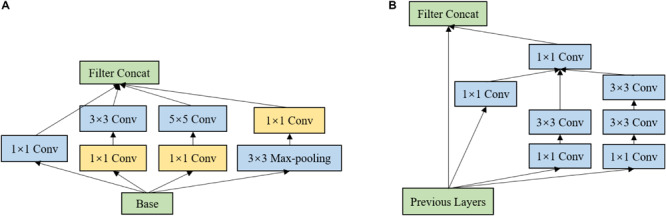
Inception structure. **(A)** Inception-v1. **(B)** Inception-ResNet v2.

The characteristics of grape leaf diseased spots are various. Black rot spots and Leaf blight spots are small and dense, while Black measles spots are similar to stripes. Thus, a single scale convolution kernel is inefficient to perceive multiscale diseased spots. To extract features of multiscale diseased spots of GLDD, Inception modules are introduced. Figure 8A shows the Inception-v1 (Szegedy et al., 2014) structure, which stacks 1 × 1 convolution layers, 3 × 3 convolution layers, 5 × 5 convolution layers, and 3 × 3 max-pooling layers, enhancing both the width of the network and adaptability of scales. Figure 8B shows the Inception-ResNet-v2 (Szegedy et al., 2016) structure, which applies the idea of residual learning to the inception network and makes it a speed boost. In the Inception modules, 1 × 1 convolution layers are inserted before or after the parallel convolution layers to reduce the thickness of feature maps and the number of weights. Inception modules can increase the depth and width of the network while reducing the number of parameters. Considering the advantages of the two inception models, the above two inception modules are brought into the backbone to improve the multiscale feature extraction capability.

### Locating and Predicting Diseased Spots

#### Locating Multiscale Diseased Spots

Region Proposal Networks are the crucial part of the detection model Faster DR-IACNN. Take Inception_5b as an example: through the pre-network, a shared feature map sizing 7 × 7 × 256 is obtained. Then, the feature map is reshaped to 7 × 7 × 512 using 3 × 3 convolution kernels. To obtain categories and regression results, the 1 × 1 convolution process is implemented in the *classification* layer and *regression* layer, obtaining feature maps of size 7 × 7 × 30 and 7 × 7 × 60, respectively. Finally, with the arranged anchors, the candidate boxes are gained.

**FIGURE 9:**
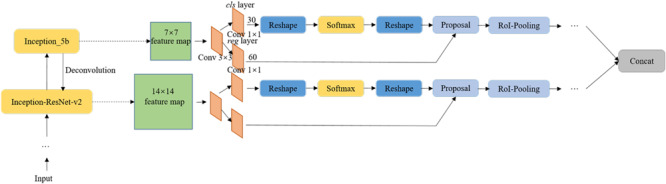
The structure of Double Region Proposal Networks.

Inspired by Feature Pyramid Networks (Lin et al., 2017), a double-RPN structure is proposed for locating the irregular and multiscale diseased spots, as shown in Figure 9. Through a deconvolution process, the high semantic information of Inception_5b is integrated with the high resolution of Inception_ResNet-v2. Thus, the proposed detection model can predict diseased spots separately in each feature layer. Furthermore, the bottom-up feature extraction and top-down upsampling method enhance the ability of the model to detect small diseased spots.

#### Guaranteeing the Translation Invariance

**FIGURE 10:**
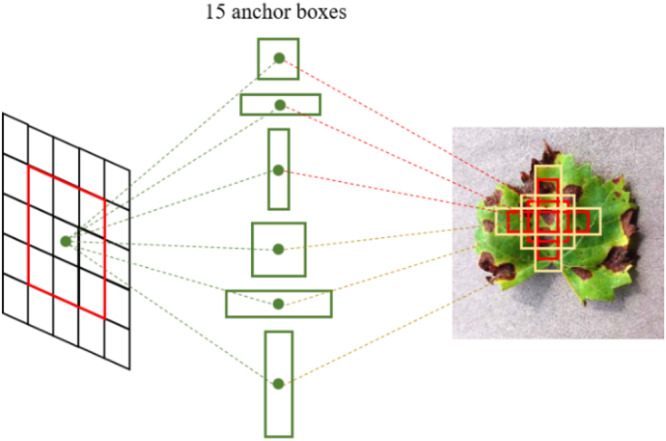
Region proposal boxes with anchors.

Due to the multiple-convolution process, the scale of feature maps is changed. It is essential to guarantee translation invariance between the feature maps and original images. To accurately locate the diseased spots on the original images, anchor boxes (Ren et al., 2018) are employed in the grape leaf diseased spot detection. As shown in Figure 10, multiple region proposals are predicted when the sliding window slides at each location. The anchor boxes’ sizes are related to scale and ratio. The default in Faster DR-IACNN is set as 5 scales and 3 ratios. That is, it generates 15 anchor boxes in the original images due to mapping. The region proposal process guarantees the essential properties of multiscale detection ability and translation invariance.

## Experimental Evaluation

This section describes the experimental setup. First, the details of the experimental platform and the dataset are introduced, and then the experimental results are analyzed and discussed.

### Experimental Setup

The experiments were performed on a 16.04.2-Ubuntu server with an Intel(R) Xeon(R) CPU E5-2650 v4 @ 2.20 GHz × 48. It is accelerated by an NVIDIA Tesla P100 PCI-E GPU, which has 3,584 CUDA cores and 16 GB of memory. The core frequency is up to 1328 MHz, and the single-precision floating-point performance is 9.3 TFLOPS. The proposed Faster DR-IACNN model was implemented on Caffe, a deep learning framework. Table 2 presents the configuration parameters.

**TABLE 2:** Hardware and software environment.

**Configuration item**	**Value**
CPU	Intel(R) Xeon(R) CPU E5-2650 v4
GPU	NVIDIA Tesla P100 PCI-E GPU 16 GB
Memory	128 GB
Hard disk	2 TB
Operating system	Ubuntu 16.04.2 LTS (64-bit)

### Dataset

In the experiment, 60% of the dataset was used for training, and the other 40% was used for validation and testing. The ratio size of the training dataset and validation dataset and testing dataset is 3:1:1. Through digit image processing technology, the number of original grape leaf disease images was expanded to 62,286. The partition of GLDD is reported in Table 3.

**TABLE 3:** Grape leaf disease dataset.

**Disease**	**Training/validation/testing image**	**Total quantity**
Black rot	9,912 / 3,304 / 3,304	16,520
Black measles	11,617 / 3,872 / 3,873	19,362
Leaf blight	9,038 / 3,013 / 3,013	15,064
Mites of grape	6,804 / 2,268 / 2,268	11,340
Total	37,371 / 12,457 / 12,458	62,286

### Experimental Results and Analyses

#### Accuracy Comparison With Various Detection Models

To compare the performance of various detection algorithms, a typical one-stage algorithm SSD and two-stage classical detection algorithms Faster R-CNN and R-FCN have been selected to detect grape leaf diseases on GLDD under the condition of using different backbone networks. The experimental results are reported in Table 4.

**TABLE 4:** Detection results of various CNN models.

**Method**	**SSD**	**R-FCN**	**Faster R-CNN**						**Faster DR-IACNN**
			
**Feature extractor**	**VGG 16**	**ResNet 50**	**ZF**	**VGG 16**	**ResNet 50**	**ResNet 34**	**ResNet 18**	**INSE-ResNet (our work)**	**INSE-ResNet (our work)**
**Classes**									
Input	512	500	500	500	500	500	500	500	500
Iterations	120 k	200 k	200 k	200 k	200 k	200 k	200 k	280 k	280 k
Black rot	74.7	**79.0**	63.5	64.5	64.4	69.3	65.8	73.7	76.7
Black measles	81.6	82.5	75.4	79.9	79.0	81.4	75.0	85.3	**88.0**
Leaf blight	72.0	68.2	59.2	60.0	60.8	64.4	64.4	71.1	**73.7**
Mites of grape	77.9	69.4	69.3	70.4	70.2	70.8	73.6	84.0	**86.2**
mAP (%)	76.6	74.8	66.9	68.7	68.6	71.5	69.7	78.5	**81.1**

*The meaning of the “bold” is the best experimental results.*

The mean Average Precision (mAP) is the standard index in the evaluation of the object detection algorithm, which is used in this section. In the two-stage algorithm, with the same input size of 500 × 500, the proposed model Faster DR-IACNN achieves a high accuracy of 81.1% mAP, and the detection performance in all categories is better than those of other detection models based on Faster R-CNN. The overall accuracy is 9.6% higher than that of the model with the backbone network ResNet34. Meanwhile, the Faster DR-IACNN model has higher detection accuracy than all of the other detection models.

To determine whether the deeper neural network can improve the detection performance of the model, ResNet50, ResNet34, and ResNet18 have been verified with 200 k iterations. The results show that ResNet50 has the worst effect, and ResNet18 did not obtain satisfactory improvement, either. The reasons for unsatisfying results are that the feature of diseased spots disappear with increasing CNN depth, while superficial layers cannot extract features accurately. Therefore, ResNet34 is the most suitable network for our dataset, with an improvement of 2.9 and 1.8% relative to ResNet50 and ResNet18, respectively.

In this experiment, Black rot and Leaf blight are relatively difficult to detect because they are similar in shape and have small diseased spots. Our proposed model, Faster DR-IACNN, shows significant improvement in the above two types of diseased spots, with 3 and 2.6% improvement compared with Faster R-CNN. Additionally, all the models have the highest detection accuracy for Black measles; the reason is that Black measles lesions are large, shaped like strips, and greatly differ from other categories, which can be easily distinguished by all the detection models.

#### Detection Accuracy and Speed

The detection speed is another important index to evaluate the object detection algorithm, which plays a vital role in real-time detection. Usually, FPS (frames per second) is utilized to evaluate detection speed. The larger the FPS is, the faster the detection speed will be. In this part, the detection speed of two-stage algorithms Faster R-CNN and R-FCN with the proposed models are evaluated. The experimental results are reported in Table 5.

**TABLE 5:** Precision and speed of various models.

**Method**	**R-FCN**	**Faster R-CNN**						**Faster DR-IACNN**
**Backbone**	**ResNet 50**	**ZF**	**VGG 16**	**GoogLeNet**	**ResNet 50**	**ResNet 34**	**ResNet 18**	**INSE-ResNet (our work)**
mAP (%)	74.8	66.9	67.5	64.3	68.6	71.5	69.7	**81.1**
Speed (FPS)	15.75	16.08	15.85	18.65	7.11	10.33	13.94	15.01

*The meaning of the “bold” is the best experimental results.*

In Faster R-CNN, the model with GoogLeNet as the backbone network has the fastest detection speed, up to 18.65 FPS. The reason is that the Inception module expends the network and reduces the parameters while deepening the network, which improves the efficiency of feature extraction. Considering the detection efficiency, the Inception module and ResNet structure have been combined to propose the Faster DR-IACNN model, which further improved the accuracy on GLDD to reach 81.1% mAP with a detection speed of 15.01 FPS, reaching the highest accuracy compared with the traditional Faster R-CNN method with a high speed that meets the actual demands in grapery. Compared with the average detection speed reached 13.66 FPS of Faster R-CNN model, which was described as a real-time detection model, the proposed Faster DR-IACNN has higher detection speed of 15.01 FPS and meets the requirements of real-time detection.

#### The Selection and Comparison of Pre-networks

The detection algorithm Faster R-CNN consists of three parts, the feature extraction network, RPN, classification and regression, among which feature extraction network (as known as a pre-network) plays an important role in the implementation of detection. In this section, several general CNNs such as GoogLeNet, VGG16, and ResNet series are trained and validated. The recognition performance of INSE-ResNet is compared with these traditional networks on GLDD. In the training process, the stochastic gradient descent (SGD) algorithm and weight attenuation strategy are adopted to minimize the loss function. In the SGD algorithm, the training image is selected randomly to update the model parameters, the batch size is set to 32, the base learning rate is 0.01, and the learning rate is attenuated three times in 10 epochs of training, which is 0.1 times of the previous learning rate such that the obtained results avoid inability to converge. To make the algorithm converge to the optimal speed, the momentum is set to 0.9.

**FIGURE 11:**
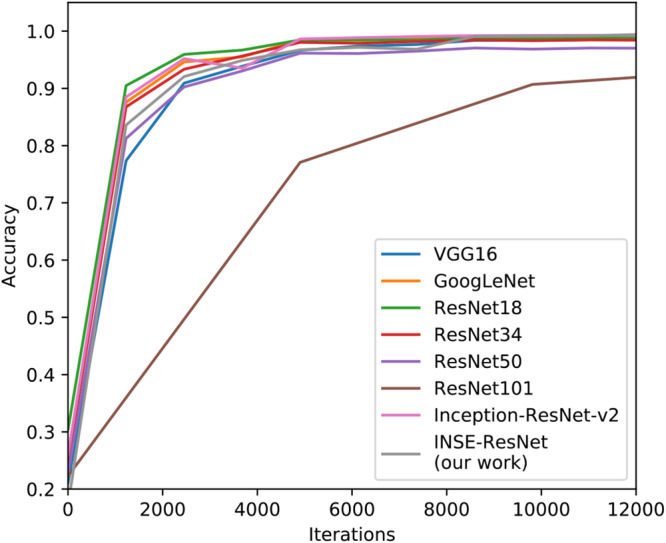
Accuracy curve of pre-networks models.

As shown in Figure 11, the X-axis represents the iterations of training, and the Y-axis represents the corresponding training accuracy. The test accuracy of each identification network in the experiments is reported in Table 6. As shown in Figure 11, the recognition results of ResNet networks and Inception networks are satisfying, which inspired us to make good use of their strengths to build INSE-ResNet. Therefore, considering the subsequent model establishment, the INSE-ResNet structure is proposed based on ResNet34 combined with the Inception-v1 module, Inception-ResNet-v2 module and SE-block, which achieves a better performance in term of recognition accuracy.

**TABLE 6:** The recognition accuracy of pre-network models.

**Pre-network model**	**Input size**	**Recognition accuracy (%)**
VGG16	224 × 224	98.48
GoogLeNet	224 × 224	98.91
ResNet18	224 × 224	98.92
ResNet34	224 × 224	98.40
ResNet50	224 × 224	97.01
ResNet101	224 × 224	88.61
Inception-ResNet-v2	224 × 224	99.28
INSE-ResNet (our work)	224 × 224	**99.47**

*The meaning of the “bold” is the best experimental results.*

#### Effect of Data Augmentation on the Detection Accuracy

To avoid overfitting, various methods are used in this article. First, the grape diseased leaves were collected in multiple environments and areas. Most diseased grape leaf images with complex background were captured in the Wei Jiani Chateau, while the other images with simpler backgrounds were collected in the laboratory, which guarantees the generalization of the proposed model and reduces the occurrence of overfitting. Moreover, due to the difficult work involved in collecting diseased grape leaves, the dataset is still insufficient. Thus, data augmentation is a satisfactory approach to solve the insufficient training image problem.

As reported in Table 7, the accuracy without data augmentation is lower, which reaches 74.3% mAP, and the loss is high during the training process. In contrast, the proposed model with data augmentation realizes 81.1% mAP, which corresponds to a detection precision improvement of 6.8% relative to the dataset without data augmentation.

**TABLE 7:** Detection precision with and without data augmentation.

**Method**	**Without data augmentation**	**With data augmentation**
Black rot	69.1	76.7
Black measles	81.4	88.0
Leaf blight	66.7	73.7
Mites of grape	80.1	86.2
mAP (%)	74.3	81.1

#### Effect of Double-RPN on the Detection Accuracy

Compared with the original RPN structure, the proposed double-RPN in Faster DR-IACNN has greatly improved the detection effect on GLDD. Double-RPN performs well at small diseased spots detection by making full use of high layers’ semantic information and low layers’ location information.

**FIGURE 12:**
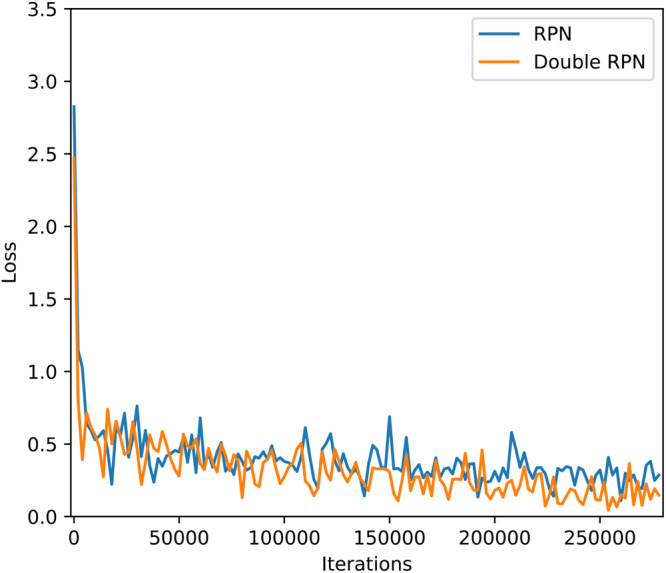
Influence of double-RPN.

This article evaluated the performance of Faster DR-IACNN on GLDD through two sets of experiments, which tested the model with original RPN and double-RPN. As shown in Figure 12, for the Faster R-CNN, the loss is relatively large and fluctuates in a large range during the training, while for the Faster DR-IACNN model, the loss function changes steadily and presents a significant downward trend. As reported in Table 8, the detection accuracy of Faster DR-IACNN for four grape leaf diseases has also improved simultaneously, and the mAP of the GLDD reaches 81.1%, increasing 2.6% compared with single RPN in Faster R-CNN.

**TABLE 8:** Detection precision with and without double-RPN.

**Methods**	**INSE-ResNet in Faster R-CNN**	**Faster DR-IACNN**
**Diseases**		
Black rot	73.7	76.7
Black measles	85.3	88.0
Leaf blight	71.1	73.7
Mites of grape	84.0	86.2
mAP (%)	78.5	81.1

#### Effect of the Anchor Scales on the Detection Accuracy

Translation invariance is a challenge of deep CNNs. Traditionally, there are two mainstream solutions to this problem: first, sampling the feature map layer in scale or aspect ratio, and second, using a filter (also considered as a sliding window) to sample in scale or aspect ratio. Faster R-CNN samples the center of convolution kernel in terms of scale and aspect ratio, which uses 3 scales and 3 aspect radios to produce 9 anchor boxes. On the feature map, RPN proposes a sliding window, whose size is 3 × 3, and takes the center of 3 × 3 sliding windows as the center of the anchor boxes.

In this article, due to the characteristic of small and dense diseased spots, the aspect ratios are set as 0.5, 1, and 2; the base size is set as 16; and the number of anchor boxes is adjusted among 3, 5, 6, and 8 for comparison. The experimental results are reported in Table 9. The results show that when the number of anchor boxes is 5 and scales are 2, 4, 8, 16, and 32—that is, the sizes of the anchor boxes are 32 × 32, 64 × 64, 128 × 128, 256 × 256, and 512 × 512—and 90 k anchor boxes are generated in each image, the highest precision of 81.1% mAP is obtained in the Faster DR-IACNN, which is 5.9% mAP higher than the original 3 anchor boxes. Especially, the detection result of small diseased spots Leaf blight is increased by 9.4% mAP.

**TABLE 9:** Evaluation of different anchor scales.

**Anchor base size**	**Number of anchors (scales)**	**Aspect ratios**	**mAP (%)**
3 scales, 3 ratios	{128^2^, 256^2^, 512^2^}	{2:1, 1:1, 1:2}	75.2
5 scales, 3 ratios	{32^2^, 64^2^, 128^2^, 256^2^, 512^2^}	{2:1, 1:1, 1:2}	**81.1**
6 scales, 3 ratios	{16^2^, 32^2^, 64^2^, 128^2^, 256^2^, 512^2^}	{2:1, 1:1, 1:2}	77.8
8 scales, 3 ratios	{32^2^, 48^2^, 64^2^, 96^2^, 128^2^, 192^2^, 256^2^, 512^2^}	{2:1, 1:1, 1:2}	78.4

*The meaning of the “bold” is the best experimental results.*

#### Detection Results of the Grape Leaves

**FIGURE 13:**
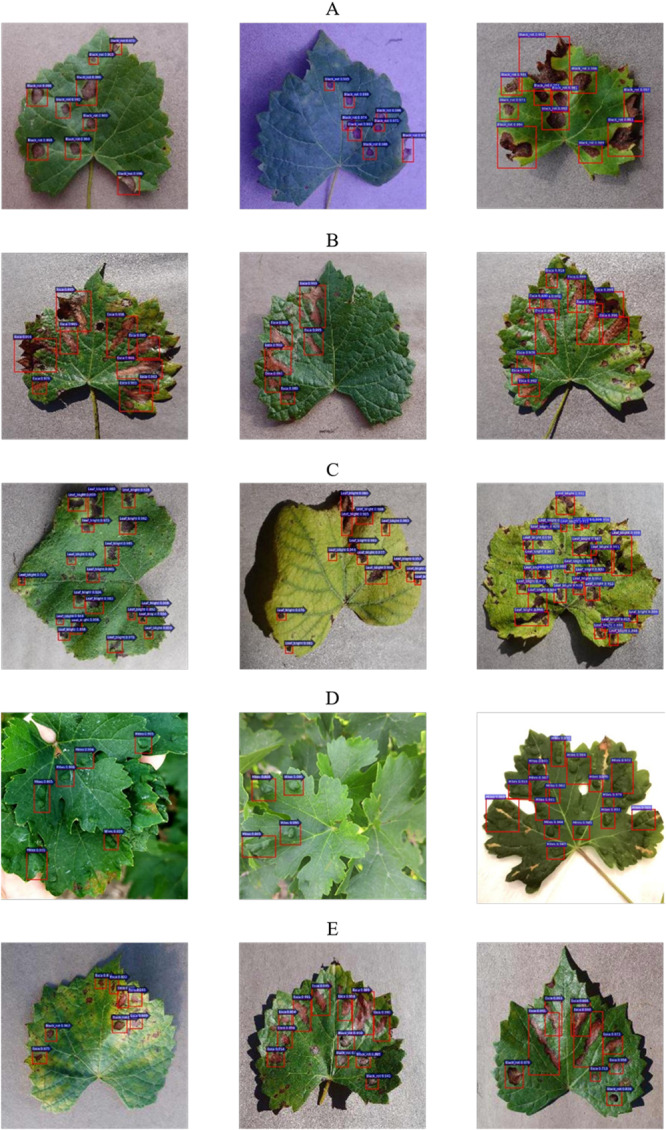
Grape leaf diseased spots detection results. **(A)** Multiple Black rot spots in one leaf. **(B)** Multiple Black measles spots in one leaf. **(C)** Multiple Leaf blight spots in one leaf. **(D)** Multiple Mites of grape spots in one leaf. **(E)** Diversified diseased spots in one leaf.

The detection results of four common diseases of grape leaves are shown in Figure 13. Figures 13A–D show the detection results of single disease of Black rot, Black measles, Leaf blight and Mites of grape, respectively, while Figure 13E shows infected leave with Black rot and Black measles simultaneously, which has been detected precisely by the detector at one time. The results show that the detection model can detect not only multiple diseased spots of the same disease in one leaf but also multiple spots of different diseases on one leaf at one time, demonstrating the strong generalization and robustness of the model. As seen in Figure 13, most scores of detection boxes are greater than 0.99, and most diseased spots on leaves are detected, demonstrating the high detection precision and accurate location of the Faster DR-IACNN model.

## Conclusion

This article proposed a deep-learning-based detector, Faster DR-IACNN, for detecting grape leaf diseases. The proposed method can automatically extract the features of diseased spots and detect four common grape leaf diseases with high accuracy and a satisfactory detection speed. To improve the generalization of the model and ensure a sufficient GLDD, 4,449 original images with both simple and complex backgrounds were collected in the laboratory and real vineyards, and a total of 62,286 diseased leaf images were generated for training via data augmentation technology. The proposed Faster DR-IACNN detector improved the detection performance of multiscale diseased spots and small diseased spots by introducing the Inception-v1 module, Inception-ResNet-v2 module and SE-blocks.

The new deep-learning-based detection method was implemented in the Caffe framework on the GPU platform. The detection performance of Faster DR-IACNN reached 81.1% mAP, and the speed was 15.01 FPS. The results demonstrate that the proposed Faster DR-IACNN method can detect four common grape leaf diseases efficiently and accurately, and it provides a feasible solution for the real-time detection of grape leaf diseases.

## Data Availability Statement

The datasets generated for this study are available on request to the corresponding author.

## Author Contributions

XX and YM contributed significantly to conducting the experiment, implementing and validating the method, writing the original draft, and revisions. BL contributed significantly to proposing the idea, providing the research project, acquiring funding, preparing, and revising the manuscript. JH, SL, and HW helped perform the analysis and provided constructive discussions.

## Conflict of Interest

HW was employed by the company West Electronic Business Co., Ltd.

The remaining authors declare that the research was conducted in the absence of any commercial or financial relationships that could be construed as a potential conflict of interest.
